# Post-infarction ventricular septal defect repair: cardioplegic arrest vs. on-pump beating-heart strategy—ten years of experience

**DOI:** 10.3389/fcvm.2025.1590588

**Published:** 2025-08-29

**Authors:** Julius Kaemmel, Leonard Pitts, Leonhard Wert, Christoph Knosalla, Miralem Pasic

**Affiliations:** ^1^ Department of Cardiothoracic and Vascular Surgery, Deutsches Herzzentrum der Charité (DHZC), Berlin, Germany; ^2^ Charité—Universitätsmedizin Berlin, Corporate Member of Freie Universität Berlin, Humboldt-Universität zu Berlin, and Berlin Institute of Health, Berlin, Germany; ^3^ DZHK (German Centre for Cardiovascular Research), Partner Site Berlin, Berlin, Germany

**Keywords:** acute myocardial infarction, post-infarction ventricular septal defect, PI-VSD, VSD, beating-heart surgery

## Abstract

**Background:**

Post-infarction ventricular septal defect (VSD) repair is in general performed on a cardioplegic heart. An alternative concept is VSD repair on a beating heart. Aim of the study was to identify possible differences in survival between the two strategies.

**Methods:**

The study was a retrospective, observational, single-centre cohort study of data from all patients who underwent post-infarction VSD repair at our institution between May 2012 and December 2022. VSD repair was performed either on an arrested heart with aortic cross-clamping [*n* = 28 (*conventional subgroup*)] or on a beating-heart [*n* = 18 (*beating-heart subgroup*)] using CPB without aortic cross-clamping. Primary end-point was survival at 1 year after surgery. Secondary endpoints included the analysis of perioperative variables, 30-day mortality and long-term survival.

**Results:**

Forty six consecutive patients underwent repair of post-infarction VSD (28 in the “*conventional subgroup”* and 18 in the “*beating-heart subgroup*”). The mean age of the patients was 66.4 ± 11.2 years and 63% were men. All-cause mortality during the first postoperative 30-days occurred in 12 of 28 patients (43%) in the “*conventional subgroup*” and in 4 of 18 patients (22%) in the “*beating-heart subgroup*”). Survival at one and five years was 34% and 27% for the “*conventional subgroup*”, compared to 72% and 61% for the “*beating-heart subgroup*”, respectively [hazard ratio, 2.3; 95% confidence interval (CI), 1.1–4.8; *P* = 0.0364].

**Conclusions:**

The modified surgical approach performing VSD-repair on a beating heart was associated with a lower risk of postoperative death than the conventional surgical strategy.

## Introduction

Post-infarction ventricular septal defect (VSD) has become rare (1). If occurring, it has devastating consequences. Post-infarction VSD repair surgery is associated with high mortality; despite adequate surgical treatment early mortality is around 40% (2, 3). Early mortality can be as high as 75% in emergency settings with patients in acute cardiogenic shock and in patients undergoing the repair soon after the causative infarction (4). Importantly, early mortality has not improved over the years (2–5). Reasons are manifold and include, among other things, a very comorbid risk profile, the rarity of the entity, and the complex surgical treatment. In order to improve the outcome for patients presenting with post-infarction VSD further advancements in the perioperative management are needed.

Change in the extracorporeal life support (ECLS) strategy, away from IABP towards ECMO and therefore the possibility of a delayed VSD repair present valuable improvements in clinical management allowing better outcomes (3, 6–8). One of the most important variables is the vulnerable myocardium in the setting of acute myocardial infarction, haemodynamic instability, low output and cardiogenic shock. Potentially, aortic cross-clamping and cardioplegic ischaemia during surgery might further aggravate this difficult situation.

Conventional surgical strategy includes VSD repair under cardioplegic arrest. An alternative is to perform a post-infarction VSD repair on the beating heart using cardiopulmonary bypass (CPB) but without cardioplegic arrest, ventricular fibrillatory arrest and without aortic cross-clamping. This strategy was adopted at our institution based on our positive experience in patients with very high surgical risk for conventional mitral valve surgery, performing mitral valve procedures with an on-pump beating-heart strategy (9). Although generally not widely popularized and accepted, different types of on-pump beating-heart techniques for different pathological conditions have already been established in some centers (10, 11). This strategy is attractive for high-risk patients with complex cardiac disease (12).

We postulated that patients with acute post-infarction VSD might have improved outcomes if the surgical repair was performed on the beating heart. We conducted a single-centre, retrospective study with data of patients who underwent post-infarction VSD repair—either on the beating heart or on the arrested heart—at our institution during the past decade. We examined perioperative data, early outcomes and long-term survival rates and subsequently focused our assessment on whether beating-heart surgery in these high-risk patients is a useful strategy.

## Methods

This study was approved by our institutional review board (Ethikkommission Charité, application number: EA2/108/22). The study complies with the Declaration of Helsinki.

### Study design

The study is a retrospective, observational, single-centre cohort study with data of all patients with acute post-infarction VSD who underwent surgical repair for this pathology at our institution during the past decade (May 2012 to December 2022). Patients were stratified according to the surgical strategy to an on-pump beating-heart VSD repair subgroup (*n* = 18) and an conventional cardioplegic arrest VSD repair subgroup (*n* = 28). The procedure was performed by the most experienced surgeons at the time of surgery. The individual treatment strategy of patients presenting to our centre with an post-infarction VSD is decided in a multidisciplinary heart team on a case-to-case basis. Beating-heart VSD repair is considered mainly for patients with proximal occlusions of the right coronary artery and in case of an earlier repair. The surgical strategy was chosen according to the surgeons preference.

The primary endpoint was one-year survival, while secondary endpoints included subgroup analysis of perioperative data, 30-day mortality, and long-term survival.

### Surgical strategy

In general, the procedure in the beating-heart subgroup is performed with the conventional surgical approach for heart surgery using CPB. Standard surgical equipment and monitoring for heart procedures is used. Transoesophageal echocardiography and ECG are performed continuously during the procedure. Carbon dioxide with a flow of 1–2 Litres is continuously insufflated into the operating field. Anaesthesia induction and maintenance are the same as for conventional heart surgery. Surgical access was in both groups via a median sternotomy. The surgery is performed alone or concomitantly with other cardiac procedures if indicated. Concomitant procedures were also performed on the beating heart, if feasible. Cardioplegia solutions used in the conventional subgroup were chosen regarding the surgeons preference and consisted of Brettschneider, Calafiore or Del Nido cardioplegia.

General rules of a beating-heart procedure: This modified technique differs from the standard surgical technique in several aspects. These aspects are crucial for the success of the procedure and are explained *in extenso* in the following text.

Access to the heart and cannulation: The heart is exposed through a median sternotomy. Like during conventional surgery, both arterial and venous cannulation are performed via the same access. There are two exceptions: cannulation of the right axillary artery or femoro-femoral cannulation if median sternotomy is planned in patients after previous heart surgery when a patent aortocoronary venous bypass (or LIMA graft) runs just behind the sternum or in patients with severe tricuspid regurgitation where the enlarged right ventricle adheres to the sternum. Then, following sternotomy and preparation of the heart (liberation from adhesions), the cannulation can be switched to standard central cannulation (*at least the venous cannula*) (9).

Cardiopulmonary bypass (CPB): The procedure is performed on normothermic partial CPB. In almost all cases it suffices to use a single two-stage (flexible) venous cannula to cannulate the right atrium. Although an air leak in the venous line could theoretically occur, this almost never happens a way that creates a problem. Total CPB (*with additional standard snaring of the superior and inferior venae cavae*) is used in additional tricuspid valve surgery or in rare situations where there is a need to access the VSD from the right ventricle for better inspection (9). To prepare for this eventuality, we routinely extend (double) the venous line already at the start of the surgery in case the superior vena cava has to be additionally cannulated during the procedure.

Placement of the left ventricular catheter (“LV vent”): An LV vent is inserted via the upper right pulmonary vein in a standard manner using a 4–0 polypropylene purse-string suture and a thin tourniquet. During the insertion, the heart is overloaded to prevent entry of air into the heart. The correct position of the vent should be verified by TEE, which should confirm that the vent has been inserted through the mitral valve and is inside the left ventricle. Importantly, the vent is fixed to the tourniquet using a free ligature only approximately 1–2 cm from the site where the vent enters the right upper pulmonary vein. This prevents unexpected vent migration (if fixed at a greater distance, the vent may migrate from the left ventricle into the left atrium). Contrary to the beating-heart mitral valve surgery where the vent suctions continuously with a flow of 2 L/min independently of the amount of blood entering the operating field (9), the LV vent during post-infarction VSD repair functions exclusively on demand. This allows all blood to be suctioned from the LV cavity, enabling excellent exposure of the LV cavity. It is possible that there is suddenly more blood in the LV cavity than expected. (*In a beating-heart procedure the aortic root is filled with blood. Any compression of the root by hand or an instrument will alter the normal anatomical relationship between the aortic valve leaflets, disrupting normal aortic valve function. The consequence is a sudden appearance of more blood in the operating field.*) The same strategy is applied in patients with preoperative aortic regurgitation of grade I-II, but additional caution is needed. The procedure is technically somewhat more challenging due to significant blood flow in the operating field. In this case, the use of an additional intracardiac sucker may be helpful (9).

De-airing of the heart: The de-airing procedure during beating-heart surgery takes significantly longer than during conventional heart surgery. Awareness of this fact is crucial for the success of the procedure. This part of the surgery must be performed very meticulously and under continuous TEE monitoring. The de-airing procedure consists of three phases: de-airing 1. of the LA and LV before tying the last stitches/sutures, 2. of the LV and 3. of the ascending aorta (9).

The first phase is performed immediately before closure of the left ventricle is completed. Simply, before tying the last two stitches, the LA vent is stopped and blood exits from the remaining opening in the suture line and the heart is de-aired by itself.

The second phase (LV de-airing) is generally performed retrogradely using an LV vent but only after filling the pericardium with saline solution. It is important that the suture line be submerged in water in order not to aspirate air into the LV cavity. (*Air can be sucked into the LV along the suture line or through the stitch holes despite LV reconstruction being completed.*) The LV vent suctions continuously with a flow of about 2 L/min while the heart is intermittently loaded and unloaded with the blood flow regulated by the perfusionist. The necessary precise commands to load and unload the heart are given loudly and clearly by the surgeon. The arterial pressure curve is monitored continuously during intermittent loading and unloading of the heart: if there are any signs of blood expulsion through the aortic valve, the heart is unloaded instantly. During loading of the heart, if TEE shows a large amount of air bubbles coming from the lung, the heart is immediately unloaded until the blood with the air bubbles has been aspirated from the left ventricle by the LV vent and no bubbles are visible inside the LV. This procedure is repeated until no more bubbles come from the lungs or are seen in the left atrium or the left ventricle. Then, at the surgeon's command, the lungs are ventilated vigorously several times by the anaesthesiologist to encourage mobilisation of potential air bubbles from the pulmonary veins. Normal ventilation of the lungs is then started. After that, the procedure of intermittent loading and unloading of the heart is repeated while the LV vent aspirates continuously with a flow of about 2 L/min (9).

The third phase is de-airing of the ascending aorta. The primary objective is to remove any accumulation of air in the proximal part of the ascending aorta in the vicinity of the right coronary ostium (air bubbles here may remain undetected by TEE) and to prevent possible air embolization into the RCA. This is achieved through a direct puncture of the very proximal part of the ascending aorta. Additionally, aortic regurgitation is intentionally induced by digital compression of the aortic base while the LV vent suctions without interruptions. TEE is continued throughout the de-airing procedure to identify any residual air in the left heart chambers. The ascending aorta is de-aired with a thin needle [e.g., 3.3-Ch needle (“yellow needle”)]. The puncture site will bleed slightly, but it is usually not necessary to suture it (9).

It should be emphasised that the de-airing procedure is performed for a period of several minutes and under continuous TEE monitoring. After LV de-airing is finished, TEE monitoring is continued to assess whether there is residual air in the heart chambers or whether any new and unexpected air bubbles are coming from the lungs or the LA or are mobilised from the LV wall (9).

Additional reperfusion of the heart and weaning from CPB: In general, slow weaning from CPB (over several minutes) is straightforward and is performed after additional reperfusion (10–20 min). It is recommended for additional recovery of the unloaded heart while the LV vent is *in situ*. It is important not to overload the heart, neither the RV nor the LV, because the RV function is usually poor. Only mild or moderate inotropic support may be necessary. Nitric oxide (NO) ventilation is added for weaning from CPB. An intra-aortic balloon pump (IABP) may be prophylactically placed in patients with a severely reduced RVEF of LVEF in order to facilitate both weaning from CPB and the postoperative course (especially during the awakening phase). After weaning from CPB, the procedure is completed in a standard manner. In some patients with a swollen heart it is recommended to leave the chest open for the following days.

“Step-by-step de-escalation of therapy”: The technique evolved during the study period mostly as a result of the more liberal use of ECMO followed by early surgery after stabilisation of organ function on ECMO. If necessary, “prophylactic” temporary RVAD implantation was considered intraoperatively or later on, at the time of ECMO explantation. (*RVAD implantation with cannulation of the peripheral vein and of the pulmonary artery using a small-diameter [usually 10 mm] Dacron graft that is tunnelled outward through the skin in the epigastrium. This enables removal of the cannula later in the postoperative course without opening the chest).* In patients on ECMO support with a critical preoperative status, we postoperatively consider a “*step-by-step de-escalation of the therapy*”, therefore a gradual reduction in intensity of all types of therapy. This includes the intraoperative consideration to leave the chest open (*both the sternum and the skin*) for 1–2 days. The main reason for this is that there is usually enormous swelling of the tissue which, if the chest were closed completely, would compress the heart. After one to two days, the next step is to close only the skin of the sternotomy wound. Definitive closure of the chest is considered 2–3 days after skin closure. The next step includes weaning the patient off ECMO, with (or without) IABP implantation at the time of ECMO explantation. If an RVAD has been implanted, a period of 10–15 days is usually needed for RV support and recovery. This is followed by tapering of the RV support and, finally, RVAD explantation. If an IABP was implanted, the last step is IABP weaning und explantation. Early tracheostomy without attempted extubation is considered in patients with a critical preoperative status or in patients of advanced age (i.e., >80 years).

Beating-heart butterfly technique: This is a novel technique reported recently (13). Briefly, the “beating-heart butterfly technique” includes closure of the ventricular septal defect with a double-layered bovine pericardial patch sewn to the intact septum. The left ventricle is opened according to the location of the post-infarction VSD in the infarct region. For an anterior VSD, the LV is incised anteriorly or through the LV apex. If the VSD is located posteriorly (inferiorly), the LV incision is made parallel to the posterior descending branch. The folded pericardial patch is first sutured close to the mitral annular level (or even to the mitral valve annulus) with deep U-stitches using 2–0 Prolene (SH needle) with pledgets (Teflon-reinforced U-stitches) sewn into the residual healthy septum. The stitches are placed from the right ventricular side of the septum —through the healthy part of the septum— into the left ventricular cavity. The sutures must be placed slowly and with the utmost precision after visualisation of all neighbouring anatomical structures. Particular attention must be paid to the atrioventricular valve and the sub-valvular structures, which are gently pulled aside by the assisting surgeon. It is important to place the U-stitches without time pressure because the heart is on-pump and unloaded. A folded xenopericardial patch is attached to the left side of the septum using interrupted sutures that are reinforced with Teflon pledgets on the right side. Placement of the U-stitches is performed under direct vision. Under no circumstances should “blind stitches” be undertaken — they are strictly prohibited. The placement of the sutures follows the shape of the post-infarction VSD (semilunar or U-shape). Depending on the size of the VSD, five to eight U-stitches are usually needed. No other sutures are placed between the patch and the endocardium of the LV chamber. The U-sutures are placed as deep as possible inside the intact myocardium, without resection of the infarcted zone. Then, the pericardial patch is folded to make it a double-layered patch. The U-stitches are placed though both layers of the doubled pericardial patch. After anchoring the patch on the left ventricular side, the patch is trimmed accordingly to achieve a semilunar or U-shape below the stitches. Usually 8–10 mm of the pericardium are left from the free ventricular edge of the pericardium to the suture row. Thereafter, continuous suturing is used to close both ventricles using one pericardial layer for closure of the LV and one layer for closure of the RV. Additional U-stitches are placed to support these continuous sutures. In this way, each layer of the doubled (folded) pericardial patch is used to close the corresponding ventricle: The double-layered pericardial patch opens up “as a butterfly”, with one side used for the reconstruction of the left ventricle and the other for the right ventricle. The heart is repositioned inside the pericardial cavity and careful de-airing is performed as explained in detail in the text above. For additional demonstration of the surgical technique see the Supplementary Video.

According to the surgeons choice, both groups were treated with the classic Daggett two-patch repair (14, 15), the Dor/David procedure (16) using a single patch for LV reconstruction or the “butterfly technique” (13) (see Supplementary Video)*.*

### Follow-up and data collection

Follow-up for the study was 100% complete. Information about deaths of patients was obtained from the hospital electronic database. All data was stored in an electronic database and subsequently analysed.

### Statistical analysis

**Figure 1:**
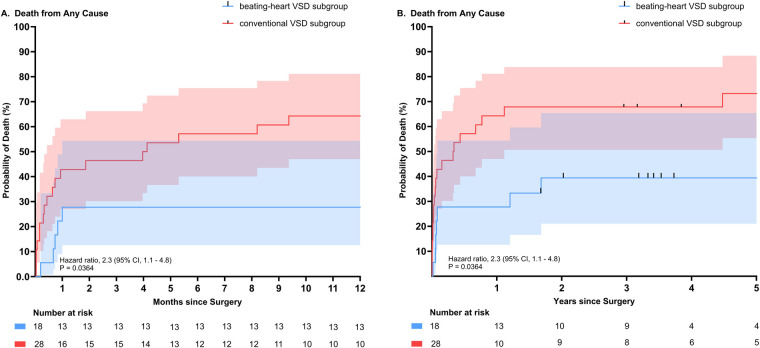
Comparison of all-cause mortality according to subgroups (beating-heart VSD repair subgroup versus conventional cardioplegic VSD repair subgroup). **(A)** All-cause mortality depicted for 1 year. **(B)** All-cause mortality depicted for up to 5 years. (Hazard ratio 2.3 (95% CI, 1.1–4.8), *P* = 0.0364).

Data was analysed in a descriptive fashion. Continuous variables were tested for normal distribution by using the Kolmogorov–Smirnov test and histograms. Normal distributed variables are presented as mean ± standard deviation or median with interquartile range (IQR), if not normally distributed. Categorical variables are presented as absolute numbers and percentages. Group characteristics and the primary endpoint 1-year survival were compared using the *t*-test, the Wilcoxon-Mann–Whitney test and the Fishers exact test for continuous, ordinal and categorical variables, respectively. Long-term survival was analyzed with the Kaplan–Meier method and Log-rank test. Two-sided *P*-values were used, with an alpha-level of 0.05. Statistical analysis was conducted using IBM® SPSS® Statistics 29 (IBM®, NY, USA). Figure 1 was created using BioRender.com and GraphPad Prism (GraphPad Software, MA, USA).

## Results

### Baseline characteristics

From May 2012 to December 2022, a total of 46 patients underwent surgery for post-infarction VSD. The analysis included all patients (28 patients in the “conventional subgroup” and 18 in the “beating-heart subgroup”). Patients characteristics and preoperative variables are presented in Tables 1–3. No significant difference was found between subgroups regarding the preoperative variables (Tables 1–3). From the whole cohort of 46 patients, 32 (69.9%) were in cardiogenic shock preoperatively. Preoperatively, 61.1% (*n* = 11) patients in the beating-heart subgroup received temporary mechanical circulatory support, compared to 53.6% (*n* = 15) in the conventional subgroup without a statistically significant difference between groups (*p* = 0.76). Intra-aortic balloon pump (IABP) was deployed in 22.2% (*n* = 4) in the beating-heart subgroup and 38.9% (*n* = 7) in the conventional subgroup. ECMO as a strategy to allow hemodynamic stabilization was used in 38.9% (*n* = 7) of patients in the beating-heart groups vs. 25% (*n* = 7) in the conventional group. One patient in the conventional group was preoperatively supported with an percutaneously placed microaxial flow pump (see Table 4).

**Table 1:** Patient demographics.

Variable	Beating-Heart subgroup (*N* = 18)	Conventional subgroup (*N* = 28)	*P*-value
Age at surgery (years)	67 ± 12	66 ± 10.9	0.772
Male gender	9 (50)	20 (71.4)	0.212
BMI (kg/m²)	28.3 (24.1–35.0)	25.4 (23.9–28.0)	0.120
EuroSCORE II (%)	18.5 (8.0–31.5)	15.4 (8.5–29.0)	0.574

**Table 2:** Preoperative cardiac function.

Variables	Beating-Heart subgroup (*N* = 18)	Conventional subgroup (*N* = 28)	*P*-value
Left ventricular ejection fraction (LVEF)			
LVEF (>50%) (normal)	3 (16.7)	13 (46.4)	0.058
LVEF (31–50%) (moderate to severely reduced)	8 (44.4)	10 (35.7)	0.758
LVEF (≤30%) (severely reduced)	7 (38.9)	5 (17.9)	0.170
Right ventricular ejection fraction (RVEF)			
RVEF (≥50%) (normal)	3 (16.7)	11 (39.3)	0.188
RVEF (40–49%) (mildly to moderately reduced)	11 (61.1)	13 (46.4)	0.378
RVEF (<30%) (severely reduced)	4 (22.2)	4 (14.3)	0.693

**Table 3:** Preoperative health status.

Variables	Beating-Heart subgroup (*N* = 18)	Conventional subgroup (*N* = 28)	*P*-value
Critical preoperative state	14 (77.8)	22 (78.6)	1
Arterial hypertension	10 (55.6)	13 (46.4)	0.763
Peripheral artery disease	2 (11.1)	2 (7.1)	0.639
Previous stroke	2 (11.1)	0 (0)	0.137
Hyperlipidemia	5 (27.8)	7 (25)	1
IDDM	1 (5.6)	3 (10.7)	1
Chronic kidney disease	2 (11.1)	4 (14.3)	1
GFR < 30 ml/min/1.73 m²	7 (38.9)	10 (35.7)	1
Regular dialysis preoperatively	0 (0)	1 (3.6)	1
Atrial fibrillation	5 (27.8)	8 (28.6)	1
Previous VSD repair	3 (16.7)	1 (3.6)	0.284
- interventional	2 (66.7)	0 (0)	0.148
- surgical	1 (33.3)	1 (100)	1
Other previous cardiac surgery	0 (0)	1 (3.6)	1
- CABG	0 (0)	1 (100)	1
One-vessel disease	7 (38.9)	11 (39.3)	1
Two-vessel disease	9 (50)	10 (35.7)	0.373
Three-vessel disease	2 (11.1)	7 (25)	0.448
Preoperative PCI	10 (55.6)	16 (57.1)	1
Anterior VSD	7 (36.8)	12 (42.9)	1
Posterior VSD	11 (61.1)	16 (57.1)	1
Time between MI and surgery			
<24 h	0 (0)	1 (3.6)	1
1–6 d	6 (33.3)	9 (32.1)	1
7–13 d	6 (33.3)	7 (25)	0.738
14–29 d	4 (22.2)	7 (25)	1
≥30 d	2 (11.1)	4 (14.3)	1
Preoperative acute kidney injury	12 (66.7)	17 (60.7)	0.761
Preoperative dialysis	1 (5.6)	4 (14.3)	0.634
Liver failure	8 (44.4)	8 (28.6)	0.347
Coagulopathy	6 (33.3)	15 (53.6)	0.232
Invasive ventilation	6 (33.3)	5 (17.9)	0.296
Pneumonia	8 (44.4)	10 (35.7)	0.758
Sepsis	0 (0)	1 (3.6)	1
Multiorgan dysfunction syndrome	7 (38.9)	5 (17.9)	0.170
Preoperative inotropes	12 (66.7)	18 (64.3)	1
Preoperative vasopressors	10 (55.6)	12 (42.9)	0.547
Preoperative cardiogenic shock	11 (61.1)	21 (75)	0.345
Preoperative CPR	1 (5.6)	1 (3.6)	1

IDDM, insulin-dependent diabetes mellitus; MI, myocardial infarction.

**Table 4:** Preoperative mechanical circulatory support (MCS).

Variables	Beating-Heart subgroup (*n* = 18)	Conventional subgroup (*n* = 28)	*P*-value
Preoperative MCS	11 (61.1)	15 (53.6)	0.763
- Preoperative IABP support	4 (22.2)	7 (38.9)	1
- Preoperative ECMO support	7 (38.9)	7 (25)	0.345
- Preoperative microaxial flow pump support	0 (0)	1 (3.6)	1

IABP, intra-aortic balloon pump; ECMO, extracorporeal membrane oxygenation.

In total, 36 (78.3%) patients were in a critical preoperative state (per EuroSCORE II definition). Twenty six patients (56.5%) underwent primary percutaneous coronary intervention prior to the procedure (Table 3). In 16 (34.8%) patients VSD repair was performed after myocardial infarction within 1–6 days, in 13 patients (28.3%) within 7–13 days and in 17 patients (37%) after more than two weeks from the initial event. In total 19 (41.3%) patients presented with an anterior VSD and 27 (58.7%) with an posterior VSD. A proximal occlusion of the right coronary artery was the culprit lesion in 12 patients (66.7%) in the beating-heart subgroup, whereas this was the case in 12 patients (42.9%) in the conventional subgroup (*p* = 0.14), respectively. The mean Qp:Qs shunt ratios were similar between the beating-heart and conventional subgroup, measured at 2.4 ± 0.8 and 2.4 ± 0.9 (*p* = 0.845), respectively.

### Intraoperative variables

Intraoperative variables are shown in Table 5. In general, there was no significant difference between subgroups. The mean cross clamp time in the conventional subgroup was 124.9 ± 47.7 min. With 13 (46.4%) cases vs. 1 (5.6%), more patients in the conventional VSD repair subgroup underwent concomitant CABG (*p* = 0.003). The “Butterfly” technique was performed more often in the beating-heart subgroup than in the conventional subgroup, with 8 (44.4%) vs. 3 (10.7%) cases, respectively (*p* = 0.014). Most patients in both groups were treated with the classical surgical VSD repair techniques. Data on intraoperative mechanical circulatory support (MCS) is presented in Table 6. Weaning from CPB was possible in all patients in the beating-heart subgroup using the described MCS strategy. In the conventional subgroup three patients could not be stabilized the discontinuation of CPB and support was terminated. These patients had prohibitive factors making use of temporary mechanical circulatory support unfeasible.

**Table 5:** Intraoperative variables.

Variables	Beating-Heart subgroup (*N* = 18)	Conventional subgroup (*N* = 28)	*P*-value
Operating time (min)	328.7 ± 121.5	392.6 ± 151.2	0.139
Perfusion time (min)	146.5 (131.3–181.8)	165.5 (136–284.8)	0.085
Concomitant CABG	1 (5.6)	13 (46.4)	**0**.**003**
Open chest therapy	6 (33.3)	7 (25)	0.738

Bold values indicate *P*-values <0.05.

**Table 6:** Intraoperative mechanical circulatory support (MCS).

Variables	Beating-Heart subgroup (*n* = 18)	Conventional subgroup (*n* = 28)	*P*-value
Intraoperative IABP implantation	3 (16.7)	5 (17.9)	1
Intraoperative ECMO implantation	2 (11.1)	4 (14.3)	1
Intraoperative ECMO explantation	5 (27.8)	4 (14.3)	0.284

IABP, intra-aortic balloon pump; ECMO, extracorporeal membrane oxygenation.

### Postoperative variables

Postoperative variables are presented in Table 7. Data on postoperative MCS is presented in Table 8. No significant differences were detected between subgroups. Frequent postoperative complications in both groups were postoperative atrial fibrillation (*p* = 1), pneumonia (*p* = 0.758) and acute kidney injury (*p* = 0.140). Due to the necessity for long postoperative mechanical ventilation, performance of a tracheotomy (*p* = 0.137) was common in both treatment groups.

**Table 7:** Postoperative variables.

Variables	Beating-Heart subgroup (*N* = 18)	Conventional subgroup (*N* = 28)	*P*-value
Duration of intensive care unit stay (d)	14.5 (6–26.25)	10 (2.75–19)	0.191
Duration of hospital stay (d)	18 (10–26.25)	12.5 (6–19)	0.105
Secondary chest closure	5 (27.9)	4 (14.3)	0.559
Rethoracotomy	3 (16.7)	10 (35.7)	0.197
Postoperative PCI	1 (5.6)	0 (0)	0.391
Sternal wound infection	2 (11.1)	1 (3.6)	0.552
Mediastinitis	0 (0)	1 (3.6)	1
Sepsis	3 (16.7)	4 (14.3)	1
Pneumonia	8 (44.4)	10 (55.6)	0.758
Tracheotomy	10 (55.6)	9 (32.1)	0.137
Cerebrovascular incident	1 (5.6)	5 (17.9)	0.380
Postoperative atrial fibrillation	11 (61.1)	18 (64.3)	1
Pacemaker implantation	1 (5.6)	2 (7.1)	1
Acute kidney injury	6 (33.3)	16 (57.1)	0.140
Postoperative dialysis	6 (33.3)	13 (46.4)	0.541
Multi organ dysfunction	4 (22.2)	9 (32.1)	0.522
Postoperative LVEF recovery^a^	15 (83.3)	18 (64.3)	0.197
Postoperative RV failure^b^	3 (16.7)	9 (32.1)	0.315
- Postoperative RV recovery^c^	2	3	0.523

^a^ LVEF increased to preoperative level if LVEF was reduced preoperatively or unchanged LVEF, if not significantly impaired preoperatively.

^b^ RVEF reduced more than 10% to preoperative RVEF or mechanical RV support or secondary organ failure due to RV failure.

^c^ RVEF increased to preoperative level if RVEF was reduced preoperatively or unchanged RVEF if not significantly impaired preoperatively.

**Table 8:** Postoperative mechanical circulatory support (MCS).

Variables	Beating-Heart subgroup (*n* = 18)	Conventional subgroup (*n* = 28)	*P*-value
Postoperative IABP implantation	0 (0)	0 (0)	
Postoperative IABP explantation	7 (38.9)	8 (28.6)	0.530
Postoperative ECMO implantation	1 (5.6)	5 (17.9)	0.380
Postoperative ECMO explantation	3 (16.7)	8 (28.6)	0.486
Postoperative microaxial flow pump implantation	0 (0)	1 (3.6)	1
Postoperative RVAD implantation	1 (5.6)	4 (14.3)	0.634
Postoperative RVAD explantation	1 (5.6)	2 (7.1)	1

### Early mortality and late survival

Survival data up to one year for both groups is illustrated and compared in Table 9. Comparison of all-cause mortality for the subgroups is illustrated and compared in Figure 1. Death from any cause during the first 30 postoperative days occurred in 12 of 28 (42.9%) patients in the conventional subgroup and in 4 of 18 (22.2%) of patients in the beating-heart VSD repair subgroup. In-hospital survival was slightly lower than 30-day survival, likely due to long duration of hospitalization, with rates of 72.2% (13/18) in the beating-heart subgroup and 50% (14/28) in the conventional subgroup (*p* = 0.220).

**Table 9:** Postoperative survival.

Variables	Beating-Heart subgroup (*N* = 18)	Conventional subgroup (*N* = 28)	*P*-value
30-day survival	14 (77.8)	16 (57.1)	0.210
3-month survival	13 (72.2)	15 (53.6)	0.234
6-month survival	13 (72.2)	12 (42.9)	0.072
1-year survival	13 (72.2)	10 (35.7)	**0**.**033**

Bold values indicate *P*-values <0.05.

One-year survival was 72.2% for the beating-heart subgroup and 35.7% for the conventional VSD repair subgroup (*p* = 0.033). With 12 patients (43.9%) vs. 3 patients (16.7%), a numerically larger proportion of patients were suffering from cardiogenic shock leading to 1-year mortality in the conventional subgroup compared to the beating-heart subgroup. The same was seen for right ventricular failure as a cause of cardiogenic shock and death during the first year after surgery, with six cases (21.4%) in the conventional subgroup vs. one case (0.6%) in the beating-heart subgroup.

Five-year survival for the beating-heart and the conventional subgroup was 61% and 27% respectively [Hazard ratio 2.3 (95% CI, 1.1–4.8), *P* = 0.0364)].

## Discussion

In our retrospective, single-institutional study involving all patients with of post-infarction VSD surgeries, the 1-year postoperative risk of death was lower among patients who underwent beating-heart surgery compared to those who had conventional surgery. Our findings suggest that beating-heart repair might improve outcomes by reducing the additional ischemic trauma from cardioplegia and aortic cross-clamping to the vulnerable myocardium.

Thirty-day mortality in the beating-heart subgroup was 22.2%, which is numerically lower than the 42.9% of the conventional subgroup. The latter was comparable to the previously reported early mortality of 37.5% at our institution when conventional surgery was performed (17). The same could be observed comparing the results of this study to large trials reporting a 30-day mortality of around 40% (3, 5). The outcome of the modified strategy presents a reduction of more than 40% in early mortality compared to our historical group (17).

A recent retrospective multi-center study by Arnoutakis et al. (18), using data from the STS database, reported a one-year survival rate of 60.9% for patients surgically treated for post-infarction VSD. This rate is comparable despite being numerically lower than the favorable survival in the beating-heart subgroup (72.2%) of our study but is higher than the conventional subgroup's survival (35.7%) which was decreased mainly by prolonged cardiogenic shock as the primary cause of death. Notably, despite earlier repairs in the cohort presented by Arnoutakis et al. (18), only 25.9% of patients were in profound cardiogenic shock, compared to markedly higher rates in both subgroups of our study. Additionally, temporary mechanical circulatory support primarily via IABP was used in 35.8% of STS patients vs. 61.1% in the beating-heart group and 53.6% in the conventional group, with many also receiving ECMO. This suggests the STS cohort may have had a more favorable preoperative status despite earlier intervention, potentially explaining the difference in survival compared to the conventional subgroup in our study.

Why does early mortality after surgical treatment of post-infarction VSD remain high?: Between the initial report by Denton Cooley et al. in 1957 and today, post-infarction VSD remains a major therapeutic challenge (19). We are still lacking a clear definition of the type of treatment (*surgical* vs. *interventional*) and the optimal timing of therapy (*early* vs. *late*). Furthermore, the risk and benefit of concomitant CABG is unclear, with large trials showing worse survival rates of patients treated with CABG and others showing no difference (3, 4, 18). At our institution, concomitant revascularization is not performed in cases where adequate preoperative revascularization has already been achieved, there is no viable myocardium at the infarction site, or the occlusions are very distal, with target vessels too small for CABG. This strategy, combined with the observation that most patients in the beating-heart group had a more proximal occlusion of the right coronary artery—typically resulting in large myocardial infarctions with less viable myocardium—might explain the difference in the rate of concomitant CABG between the groups.

The early mortality associated with this difficult surgical problem remains high. One limiting factor is that post-infarction VSD is extremely rare in comparison with other cardiac pathologies. It is almost impossible for a single institution, let alone a single cardiac surgeon, to gain any amount of experience that would lead to improved results. Notably, by treating about five post-infarction VSD repair cases annually, the presented cohort is relatively large compared to an annual rate of three cases per surgical center in a large multicentric study (4). Possible drawbacks are that some patients are declared as “inoperable” even if this is not truly the case. Furthermore, the refusal to attempt to treat the most difficult cases by surgical means, possibly with worse results, pushes alternative therapies, such as interventional closures with an occluder (20). Although survival of the group as a whole is suboptimal, we must not forget that almost all of these patients would not survive without surgery (21).

Rationale for on-pump beating-heart post-infarction VSD surgery: Theoretical advantages have been described elsewhere (9). In summary, this strategy dispenses with aortic cross-clamping and cardioplegia and is therefore expected to eliminate additional ischaemic trauma to the heart (22). Cardioplegic cardiac arrest is a major cause of postoperative morbidity in patients with left ventricular dysfunction (22). Compared to patients with cardioplegic arrest, those undergoing beating-heart mitral valve surgery had lower postoperative creatine kinase-MB levels and a shorter period of postoperative inotropic support (23). In the setting of post-infarction VSD, where patients typically present in beginning or established cardiogenic shock, avoidance of any additional myocardial injury is of utmost importance. As previously reported, beating-heart VSD surgery was generally considered in patients with a more proximal occlusion of the right coronary artery or in cases requiring earlier repair despite use of mechanical circulatory support, both of which theoretically increase the likelihood of postoperative heart failure. This aligns with the finding of lower postoperative mortality in the beating-heart subgroup compared to the conventional subgroup, where mortality was predominantly attributed to postoperative cardiogenic shock.

Weaning from CPB was successful in all patients in the beating-heart subgroup. Therefore, this modification is particularly important for uneventful weaning from the heart-lung machine. Furthermore, the beating-heart technique can be used for anterior and posterior VSD in the same way as conventional surgery.

## Conclusion

In our retrospective, single-institutional study involving all our patients operated on for postinfarction VSD, the use of the beating-heart strategy was associated with lower all-cause mortality at one year compared to the conventional strategy with cardioplegic arrest. The modified on-pump beating-heart technique may reduce intraoperative factors for immediate heart failure, potentially enabling better and easier weaning from CPB. On-pump beating-heart repair for post-infarction VSD should be considered an additional tool in the surgical armamentarium.

## Limitations

Our study has a number of important limitations. It covers only a small number of patients, which limits statistical analyses and detection of bias. Furthermore, the retrospective and unicentric nature of the study may impact the generalizability of the results. The difficulty in assessing the appropriate place of this new therapy in a rigorous scientific fashion is attributable to the low incidence of post-infarction VSD. A control-randomized trial is indicated but it is not feasible to perform such a trial in this particular surgical group of patients. Our study group included a wide range of patients in whom not only the location and the size of the post-infarction VSD but also the surgical risks and grades of comorbidities varied. Despite the apparent differences, these subgroups of patients were all united by the fact that they were treated with the same surgical strategy — either the conventional or the modified surgical technique. Furthermore, our general strategy evolved over time due to the more liberal use of ECMO in the last years, which allows the recovery of organ function and may influence the outcomes. Although post-infarction VSD repair was in our study performed by the most experienced cardiac surgeons, patient outcomes may still be influenced by individual surgeon experience and technique.

## Data Availability

The original contributions presented in the study are included in the article/Supplementary Material, further inquiries can be directed to the corresponding author.
