# Supplementary material for: Post-infarction ventricular septal defect repair: cardioplegic arrest vs. on-pump beating-heart strategy—ten years of experience
Source: Front Cardiovasc Med. 2025 Aug 29;12:1590588. doi: 10.3389/fcvm.2025.1590588 (PMC12426030; doi:10.3389/fcvm.2025.1590588)
Supplement: Supplementary file 1 [file Datasheet1.pdf]

Supplementary Material

Post-infarction ventricular septal defect repair:

cardioplegic arrest versus on-pump beating-heart strategy

– ten years of experience –

Julius Kaemmel MD, Leonard Pitts MD, Leonhard Wert MD, Miralem Pasic MD

Deutsches Herzzentrum der Charité

Berlin, Germany

1 Supplementary Data

Timing of surgery – early/immediate versus late treatment

The data published thus far suggest that the optimal timing of surgery is not yet established. European guidelines (1, 2) recommend early surgery in all patients with severe heart failure who fail to respond rapidly to aggressive therapy. However, early treatment is still associated with a high mortality despite prompt treatment (3-7). The guidelines propose a delayed elective surgical repair in patients who respond well to aggressive heart failure therapy (1, 2). Although delayed surgery allows easier septal repair with matured scar tissue, it bears the risk of abrupt haemodynamic deterioration and death while waiting for surgery (1, 2). The time of prompt deterioration of the clinical situation after initial stable situation is not known. Sander et al. published in 1956 that due to a natural course (i.e. no surgery) of the pathological feature, 54% of the patients die within 7 days and 89% within 60 days (8). The modified operative strategy described in this paper, with on-pump beating-heart repair of a post-infarction VSD, practically enables "early surgery" immediately after acute myocardial infarction without an increase —or perhaps even with a decrease— in early mortality.

In the early era of heart surgery the operative strategy, as Daggett et al. summarised nicely many years ago, was that "*most of these procedures were conducted in patients several weeks to months after septal rupture, in individuals who had completely recovered from their infarction*"(9). Due to natural selection during the waiting time and healing of the tissue neighbouring the VSD, these patients —both today and in the past— would fall into a low-risk group (6, 9). This strategy resulted in a single centre with a low mortality already some 35 years ago (10). In 1990 Skillington et al. published their experience in a total of 101 patients (mean age 64.9 years) who underwent surgical correction of post-infarction VSD at Wessex Cardiothoracic Centre, Southampton General Hospital, England, over a 15-year period (1973 to 1988) (10). The overall early mortality rate was found to be 20.8%, although this figure decreased to 11.1% based on experience in 36 patients from January 1987 to October 1988 (10). The majority of other centres were unable to reproduce these outcomes. In a study published in 2008, in-hospital death was higher among patients who underwent early surgery (≤5 days) than in those who

underwent late surgery (> 9 days) (73% versus 27%, respectively) (7). The largest study published so far is a US study with 2876 adults who underwent post-myocardial infarction VSD repair. This was a retrospective review of the Society of Thoracic Surgeons' National Database covering the period between 1999 and 2010. The analysis showed a high operative mortality rate of 42.9% in the overall cohort (5). The rate was considerably higher (54.1%) if the repair was performed within 7 days of a myocardial infarction, compared to an early mortality of only 18.4% if than more than 7 days had passed since the myocardial infarction (5). These results were confirmed by the recently published representative "CAUTION Study" (4), the largest multicentre observational study with 475 patients from 26 different centres worldwide. The study included procedures performed between January 2001 and December 2019. It showed that delayed surgery may be associated with better survival. The study demonstrated that the early mortality rate for surgically treated ventricular septal rupture was 40.4%, mostly due to a low cardiac output (4). The UK National Adult Cardiac Surgery Audit database report, also recently published, analysed operative mortality in 1010 patients undergoing surgical VSD repair from 1996 to 2018 (6). Operative mortality was 38.9% overall and was higher when patients underwent surgery within the first 6 hours (75%) or the first 24 hours (61.3%) from the index myocardial infarction. Both the number of surgical VSD repairs and the mortality rate did not change significantly over the 23-year period (6). Interestingly, data published by Jaswaney et al. showed that early repair of post-infarction VSD in patients with cardiogenic shock was associated with a lower mortality (11). Conversely, in patients without cardiogenic shock, early repair was associated with a higher mortality (11).

Alternative therapies

More recently interventional catheter-based percutaneous post-infarction VSD closure has emerged as a treatment alternative. Most commonly used for interventional closure of post-infarction VSD are the Amplatzer™ occluder devices (Abbot Laboratories, IL, USA)). Despite advancements in percutaneous VSD closure techniques since their first introduction, short- and long term mortality of patients remains high. In a large multicentric retrospective UK registry study conducted by Giblett et al. comparing outcomes of interventional post-infarction VSD closure and surgical VSD repair, the in-hospital mortality rate was with 55% versus 44.2% (p=0.048) significantly higher in the interventional group and comparable at 5 years (61.1% versus 53.7%; p=0.65) (12). Data published thus far suggest that the optimal timing of intervention with a device, or the optimal patient population for the intervention, has not yet been established due to a lack of more extensive experience (13). Another interesting strategy is to initially treat small or medium post-infarction VSDs with a ventricular septal occluder to stabilise patients and allow myocardial fibrosis, thus facilitating a delayed surgical correction (14). In case all treatment strategies fail, the last therapeutic option is excision of the heart and implantation of the total artificial heart.

References

1. Byrne RA, Rossello X, Coughlan JJ, Barbato E, Berry C, Chieffo A, et al. 2023 ESC Guidelines for the management of acute coronary syndromes: Developed by the task force on the management of acute coronary syndromes of the European Society of Cardiology (ESC). European Heart Journal. 2023;44(38):3720-826.
2. Ibanez B, James S, Agewall S, Antunes MJ, Bucciarelli-Ducci C, Bueno H, et al. 2017 ESC Guidelines for the management of acute myocardial infarction in patients presenting with ST-segment elevation: The Task Force for the management of acute myocardial infarction in patients presenting with ST-segment elevation of the European Society of Cardiology (ESC). European Heart Journal. 2017;39(2):119-77.

3. Matteucci M, Ronco D, Corazzari C, Fina D, Jiritano F, Meani P, et al. Surgical Repair of Postinfarction Ventricular Septal Rupture: Systematic Review and Meta-Analysis. Ann Thorac Surg. 2021;112(1):326-37.
4. Ronco D, Matteucci M, Kowalewski M, De Bonis M, Formica F, Jiritano F, et al. Surgical Treatment of Postinfarction Ventricular Septal Rupture. JAMA Netw Open. 2021;4(10):e2128309.
5. Arnaoutakis GJ, Zhao Y, George TJ, Sciortino CM, McCarthy PM, Conte JV. Surgical repair of ventricular septal defect after myocardial infarction: outcomes from the Society of Thoracic Surgeons National Database. Ann Thorac Surg. 2012;94(2):436-43; discussion 43-4.
6. Dimagli A, Guida G, Sinha S, Dixon L, Fudulu D, Gemelli M, et al. Surgical outcomes of post-infarct ventricular septal defect repair: Insights from the UK national adult cardiac surgery audit database. J Card Surg. 2022;37(4):843-52.
7. Figueras J, Alcalde O, Barrabés JA, Serra V, Alguersuari J, Cortadellas J, et al. Changes in hospital mortality rates in 425 patients with acute ST-elevation myocardial infarction and cardiac rupture over a 30-year period. Circulation. 2008;118(25):2783-9.
8. Sanders RJ, Kern WH, Blount SG, Jr. Perforation of the interventricular septum complicating myocardial infarction; a report of eight cases, one with cardiac catheterization. Am Heart J. 1956;51(5):736-48.
9. Daggett WM. Postinfarction ventricular septal defect repair: retrospective thoughts and historical perspectives. Ann Thorac Surg. 1990;50(6):1006-9.
10. Skillington PD, Davies RH, Luff AJ, Williams JD, Dawkins KD, Conway N, et al. Surgical treatment for infarct-related ventricular septal defects. Improved early results combined with analysis of late functional status. J Thorac Cardiovasc Surg. 1990;99(5):798-808.
11. Jaswaney R, Arora S, Khwaja T, Shah N, Osman MN, Abu-Omar Y, et al. Timing of Repair in Postinfarction Ventricular Septal Defect. Am J Cardiol. 2022;175:44-51.
12. Giblett JP, Matetic A, Jenkins D, Ng CY, Venuraju S, MacCarthy T, et al. Post-infarction ventricular septal defect: percutaneous or surgical management in the UK national registry. Eur Heart J. 2022;43(48):5020-32.
13. Cadogan D, Daghem M, Snosi M, Williams LK, Weir-McCall J, Calvert PA, et al. Percutaneous Transcatheter Closure of Post-infarction Ventricular Septal Defect: An Alternative to Surgical Intervention. Interv Cardiol. 2023;18:e19.
14. Maltais S, Ibrahim R, Basmadjian AJ, Carrier M, Bouchard D, Cartier R, et al. Postinfarction ventricular septal defects: towards a new treatment algorithm? Ann Thorac Surg. 2009;87(3):687-92.

1.1 Supplementary Video

The supplementary video demonstrates the on-pump beating heart-strategy in the management of a posterior post-infarction ventricular septal defect. The video highlights key procedural steps.
